# Experimental and modeling evidence of carbon limitation of leaf appearance rate for spring and winter wheat

**DOI:** 10.1093/jxb/erz012

**Published:** 2019-02-20

**Authors:** Maeva Baumont, Boris Parent, Loïc Manceau, Hamish E Brown, Steven M Driever, Bertrand Muller, Pierre Martre

**Affiliations:** 1 LEPSE, Université Montpellier, INRA, Montpellier SupAgro, Montpellier, France; 2 The New Zealand Institute for Plant & Food Research Limited, Private Bag, Christchurch, New Zealand; 3 Centre for Crop Systems Analysis, Department of Plant Sciences, Wageningen University, AK Wageningen, The Netherlands

**Keywords:** Carbon, crop model, daylength, leaf appearance rate, photoperiod, photothermal quotient, phyllochron, *SiriusQuality*, temperature, wheat

## Abstract

Accurate predictions of the timing of physiological stages and the development rate are crucial for predicting crop performance under field conditions. Plant development is controlled by the leaf appearance rate (LAR) and our understanding of how LAR responds to environmental factors is still limited. Here, we tested the hypothesis that carbon availability may account for the effects of irradiance, photoperiod, atmospheric CO_2_ concentration, and ontogeny on LAR. We conducted three experiments in growth chambers to quantify and disentangle these effects for both winter and spring wheat cultivars. Variations of LAR observed between environmental scenarios were well explained by the supply/demand ratio for carbon, quantified using the photothermal quotient. We therefore developed an ecophysiological model based on the photothermal quotient that accounts for the effects of temperature, irradiance, photoperiod, and ontogeny on LAR. Comparisons of observed leaf stages and LAR with simulations from our model, from a linear thermal-time model, and from a segmented linear thermal-time model corrected for sowing date showed that our model can simulate the observed changes in LAR in the field with the lowest error. Our findings demonstrate that a hypothesis-driven approach that incorporates more physiology in specific processes of crop models can increase their predictive power under variable environments.

## Teaser

Carbon availability can account for environmental effects on leaf appearance rate (LAR) and a new ecophysiological model is developed that gives good predictions for LAR in contrasting field environments.

## Introduction

The rate at which plants develop strongly affects canopy and root structure, radiation interception, and, through the cumulative effects of these factors, biomass production, partitioning, and yield. It is therefore essential to understand how this rate is determined and how it can be modeled in order to accurately predict crop responses to their environment in the field. A widely used metric to quantify plant development rate is the phyllochron, i.e. the time-interval between successive organs at the same stage (Wilhelm and McMaster, 1995), or its reciprocal, the leaf appearance rate (LAR). Both are often expressed in (or per) thermal-time unit (i.e. in cumulative temperature above a base temperature, classically expressed in degree-days, °Cd). The phyllochron has been used for decades in the plant science community and many growth simulation models use it to model both vegetative and reproductive development (Rickman and Klepper, 1995; Fournier *et al.*, 2005; Evers *et al.*, 2006).

The success of the phyllochron as a straightforward concept relies on the linear relationship between LAR and temperature, and therefore its constancy when expressed in thermal time. However, in many situations, irregular or non-linear relationships between leaf appearance and temperature limit its value to predict development. In several grasses, including wheat, LAR increases with photoperiod (Baker *et al.*, 1980; Cao and Moss, 1989*a*; Masle *et al.*, 1989; Slafer *et al.*, 1994), irradiance (Rickman *et al.*, 1985; Volk and Bugbee, 1991; Bos and Neuteboom, 1998; Birch *et al.*, 1998), and atmospheric CO_2_ concentration (Boone *et al.*, 1990; McMaster *et al.*, 1999), whilst it decreases with plant density (Abichou *et al.*, 2018), red/far-red ratio and blue light (Gautier and Varlet‐Grancher, 1996), and nitrogen or water deficit (Longnecker and Robson, 1994).

LAR is also often reported to change with ontogeny. Indeed, the relationship between the number of visible leaves and thermal time appears as either bilinear or non-linear, both under fluctuating field conditions (Baker *et al.*, 1986; Hay and Delécolle, 1989) and under constant controlled conditions (Cao and Moss, 1991; Slafer and Rawson, 1997; Miralles and Richards, 2000). Changes in LAR with ontogeny could be related to an increase in the time taken by successive leaves to extend above the whorl of previous leaves (Miglietta, 1991; Skinner and Nelson, 1995; Streck *et al.*, 2003). However, LAR for wheat increases and decreases with leaf rank for late and early sowing, respectively, independently of sheath length (Hay and Delécolle, 1989; Abichou *et al.*, 2018). An alternative hypothesis is that the phyllochron changes with specific developmental stages. In wheat, ontogenic changes in LAR coincide with the initiation of the flag-leaf primordium (Abichou *et al.*, 2018) or first-ridge formation (Boone *et al.*, 1990). However, in other cases, ontogenic changes in LAR occur around the time of appearance of a given leaf, independently of final leaf number and of the state of the apex (Slafer and Rawson, 1997), which suggests that ontogenic changes in LAR are not associated with any particular growth stage. Finally, it has been suggested that, at least in some conditions, apparent ontogenic changes in LAR might be due to the use of an incorrect base temperature (Hay and Delécolle, 1989).

Several models accounting for the effects of temperature and photoperiod on LAR have been proposed and compared with each other (Miglietta, 1991; Kirby, 1995; Bindi *et al.*, 1995; McMaster and Wilhelm, 1995). However, with the exception of Miglietta (1991), these models do not consider changes in LAR with ontogeny. Surprisingly, none of these LAR models have been incorporated into crop growth models, where LAR is modeled predominantly as a linear response to temperature, without any effects of photoperiod or plant age (Muchow and Carberry, 1990; Amir and Sinclair, 1991; Lizaso *et al.*, 2011). Only a few crop growth models consider photoperiod or plant age effects on LAR. For instance, the wheat model Sirius uses three different constant LAR values depending on leaf rank (Jamieson *et al.*, 1995, 1998), and the photoperiod effect is modeled using an empirical relationship between sowing date and LAR (He *et al.*, 2012). A similar approach is used in the APSIM-NWheat model, where the phyllochron is empirically corrected at a fixed date after sowing (Bassu *et al.*, 2009). A recently updated version of APSIM wheat (Brown *et al.*, 2018) models phyllochron as a function of leaf rank and a photoperiod adjustment factor.

In this study, we tested the hypothesis that carbon availability could account for the effects of temperature, irradiance, photoperiod, air CO_2_ concentration, and ontogeny on LAR. This hypothesis fits well with most of the effects noted above, such as the positive effect on LAR of the photoperiod or of elevated CO_2_, as well as the negative effect of elevated temperature, which decreases the amount of fixed carbon per unit thermal time. Changes in LAR with ontogeny could be related to the strong alterations of source–sink relationships that take place during development (Dingkuhn *et al.*, 2005). Moreover, carbon status, in particular in the lower range, is often reported as driving shoot development (Masle, 2000; Stitt and Zeeman, 2012). Finally, carbon status is often reported to alter LAR in woody species (e.g. Davidson *et al.*, 2016).

We conducted three experiments in growth chambers in order to quantify and disentangle the effects of temperature, light intensity, photoperiod, air CO_2_ concentration, and ontogeny for both winter and spring wheat cultivars. The photothermal quotient (PTQ, mol m^−2^ °Cd^−1^), defined as the ratio between daily photosynthetically active radiation (PAR, mol m^−2^ d^−1^) and mean daily thermal time, was used to quantify the (potential) supply of carbon per unit of development time. Because our experimental results showed good agreement with our hypothesis, we developed a simple ecophysiological model that accounts for temperature, light, and photoperiod effects, as well as the effects of ontogeny on LAR. This model was integrated in the wheat model *SiriusQuality* (Martre *et al.*, 2006; He *et al.*, 2012; Wang *et al.*, 2017). Comparisons of leaf stages simulated with our model or with either a simple linear model or the current LAR model of *SiriusQulity* against field data with a very large range of daily mean temperatures and photoperiods showed that our proposed model accurately simulated the observed changes in LAR with sowing date (photoperiod), temperature, and ontogeny.

## Materials and methods

### Plant material and growth conditions

Three independent experiments were carried out on wheat (*Triticum aestivum*) under controlled environment conditions using winter and spring cultivars (Table 1, Supplementary Table S1 at *JXB* online). The first experiment studied the response of leaf appearance rate (LAR) to different combinations of temperature, irradiance, and photoperiod; the second studied the response of LAR to elevated CO_2_ at two temperatures; and the third studied the genetic variability of the response of LAR to the photothermal quotient (PTQ, mol m^−2^ °Cd^−1^).

**Table 1.** Cultivars and environmental conditions for the three experiments carried out in this study

Experiment/ Cultivar^a^	Treatment name	Set point day/night air temperature (°C)	Set point PAR (µmol m^−2^ s^−1^)	Photoperiod (h)	Air CO_2_ concentration (ppm)	Mean daily PAR (mol m^−2^ d^−1^)	Mean daily thermal time (°Cd)	Photothermal quotient (mol m^−2^ °Cd^−1^)	Mean day/night leaf–air VPD (kPa)	LAR_i_ (×10^−3^ leaves °Cd^−1^)
Experiment 1. Photothermal effect										
*Paragon*, ***Renan***, **Récital**	HT.SD.320	28/24	320	8	400	9.4	24.5	0.38	1.7 /1.1	6.61±0.37
	HT.LD.170	28/24	170	16	400	9.6	26.2	0.37	1.6 /1.0	6.63±0.47
	HT.LD.280	28/24	280	16	400	16.5	26.2	0.63	1.5/0.9	7.94±0.22
	LT.LD.280	18/14	280	16	400	15.9	10.3	1.54	0.8/0.6	10.84±0.52
	HT.MD.450^b^	28/24	450	14	400	22.5	26.4	0.85	1.7 /1.1	7.75±0.83
	LT.MD.320^b^	18/14	320	14	400	15.7	10.5	1.50	1.12/0.8	9.17±0.71
	280→170^c^	28/24	280→170	16	400	16.5→9.6	26.2→26.2	0.63→ 0.37	1.5/0.9→1.6 /1.0	7.80±0.52
	170→280^c^	28/24	170→280	16	400	9.6→16.5	26.2→26.2	0.37→0.63	1.6 /1.0→1.5/0.9	6.49±0.43
Experiment 2. CO_2_×temperature effect										
*Paragon*	HT.aCO_2_	28/24	600	14	400	32.3	27.2	1.19	1.1 /1.0	10.80±0.56
	HT.eCO_2_	28/24	600	14	800	27.8	27.2	1.02	1.1 /1.0	11.90±0.64
	LT.aCO_2_	18/14	600	14	400	31.5	10.6	2.97	1.0 /0.9	11.20±1.19
	LT.eCO_2_	18/14	600	14	800	30.0	10.6	2.83	1.1/1.1	15.80±0.83
Experiment 3. Genetic variability										
Apache-sp, *Arche*, Baviacora M92, *Cadenza*, Chinese Spring, *Courtot*, Drysdale, Feeling, Gladius, *Paragon*, Récital-sp, Seri M82, *Specifik*, Yecora Rojo, Yitpi	HT.SD	28/24	190	8	400	4.9	25.5	0.20	1.48/0.84	5.00±0.39
	HT.LD	28/24	190	16	400	11.1	26.7	0.42	1.52/0.91	6.62±0.54
	LT.SD	18/14	190	8	400	5.7	8.9	0.64	1.57/0.92	6.58±0.42
	LT.LD	18/14	190	16	400	10.6	10.1	1.05	1.43/0.87	8.40±0.59

Details of the 17 cultivars used are given in Supplementary Table S1. Initial leaf appearance rates (LAR_i_) are given for the spring wheat cv. Paragon, which was grown in all experiments and treatments. LAR_i_ data are mean (±1SD) for *n*=4–6 independent replicates. VPD, vapour-pressure deficit.

^a^ Italics, photoperiod sensitive cultivars; bold, winter wheat cultivars.^b^ Treatments were only applied to the spring wheat cv Paragon.^c^ Treatments were only applied to the winter wheat cv. Récital.

In all experiments, seeds were imbibed for 24 h at 4 °C on wet filter paper in Petri dishes, then placed at room temperature (22 °C) for 24 h, and transferred back to 4 °C until the radicles were 5 mm long. In Experiments 1 and 3, uniform-sized seedlings were then transplanted into 1.7-L plastic pots (one plant per pot) filled with a 30:70 (v:v) mixture of soil and organic compost. Pots were placed in controlled environment growth chambers with different conditions but with a day/night air vapor-pressure deficit of 1.5/1.0 kPa set as common to all experiments and treatments. Each growth chamber was associated with one treatment, representing a combination of temperature, light intensity, and photoperiod, as detailed in Table 1. In Experiment 1, treatments 280→170 and 170→280 consisted of a swap between growth conditions when plants had 3.5 visible leaves. Six independent replicates were used in each treatment and the genotypes were randomized in the growth chambers. Plants were watered daily and no nutrients were applied as the potting substrate provided enough to the plants for the duration of the experiments.

In Experiment 2, uniform-sized seedlings were transplanted to 3-l plastic pots (one plant per pot) filled with soil. Fifteen plants of each cultivar per growth temperature were placed in a five-block design in two walk-in growth chambers. In both chambers, the air vapor-pressure deficit was maintained constant at 1.0 kPa. Plants were watered daily and additional nutrients were supplied by watering with 300–500 ml of Hoagland solution (Hoagland, 1950) three times per week, from 3 weeks after transplanting.

In all experiments, leaf (*T*_leaf_) and apex (*T*_apex_) temperatures (°C) were measured with thermocouples secured on the lower surface of leaf blades or inserted vertically between leaf sheaths down to the base of the leaves, respectively.

### Determination of leaf appearance rate

Main stem leaf stages were determined every2–3 d from the ligulation of the second leaf to the appearance of the flag-leaf ligule for the spring cultivars or to the ligulation of leaf 10 for the winter cultivars. The Haun stage (Haun, 1973) was calculated as the ratio of the length of the youngest visible (expanding) leaf blade to the length of the blade of the youngest ligulated (mature) leaf. The initial LAR (LAR_i_) was calculated as the slope of the relationship between the Haun stage and thermal time calculated using the apex temperature for Haun stage ≤5 to avoid confounding effects due to the increase in final leaf length after leaf 5 (Martre and Dambreville, 2018). To assess the changes in LAR over plant development, a spline function was fitted to Haun stage versus thermal time and LAR was calculated by taking the first derivative of the fitted spline equations.

The daily thermal time (∆*T*_t_, °Cd) was calculated as:

$$\begin{array}{l} \Delta T_{t}=\underset{i=1}{\overset{n}{\sum }}\;\frac{1}{144}\underset{i=1}{\overset{n=144}{\sum }}\;T_{opt}\times f\left(T\right) \end{array}$$ (1)

with

$$\begin{array}{ll} f\left(T\right)=\; & max\left(0,\frac{2{\left(T_{apex}-T_{min}\right)}^{\alpha }{\left(T_{opt}-T_{min}\right)}^{\alpha }-{\left(T_{apex}-T_{min}\right)}^{2\alpha }}{{\left(T_{opt}-T_{min}\right)}^{2\alpha }}\right);\;\\ & \alpha =\frac{ln2}{ln\left(\frac{T_{max}-T_{min}}{T_{opt}-T_{min}}\right)} \end{array}$$ (2)

Where *f*(*T*) (dimensionless) is the non-linear temperature response of leaf initiation and growth (Wang *et al.*, 2017), *T*_apex_ is the 10-min mean apex temperature, and *T*_min_, *T*_opt_, and *T*_max_ are the minimum, optimum, and maximum temperatures, respectively. Values of 0, 27.5, and 40 °C were used for *T*_min_$T_{min}$, *T*_opt_, and *T*_max_, respectively (Parent and Tardieu, 2012; Wang *et al.*, 2017). The photothermal quotient (mol m^−2^ °Cd^−1^) was calculated as the ratio of daily PAR to ∆*T*_t_ (Nix, 1976).

### Gas-exchange measurements

In Experiment 1, net carbon assimilation (*A*_net_, µmol CO_2_ m^−2^ s^−1^) was measured for cv. Paragon on leaf 3 the day after its ligulation using a CIRAS-2 portable photosynthesis system (PP Systems, Amesbury, MA, USA) equipped with a 25×7-mm bead plate. Measurements were carried out under ambient temperature (leaf temperature set equal to ambient air temperature), light intensity (provided by red-white LEDs), and air CO_2_ concentration (400 ppm). The cuvette relative humidity was set to maintain the ambient air vapor-pressure deficit. Daily carbon assimilation (*A*_day_, mol CO_2_ m^−2^ °Cd^−1^) was calculated by integrating *A*_net_ over the diurnal period.

 In Experiment 2, assimilation at saturating light intensity (PAR=1600 µmol m^−2^ s^−1^; *A*_sat_) was measured instead of *A*_net_ on leaf 4 with a LI-6400XT portable photosynthesis system (LI-COR, Lincoln NE, USA) fitted with a 6400–40 Leaf Chamber Fluorometer. Light was provided by a red-blue LED light source (10% blue light), leaf temperature was maintained near growth temperature (18 or 24 ±1 °C), the CO_2_ concentration of the air was maintained near growth CO_2_ (400 or 800 ppm), and the air–leaf vapor-pressure deficit was maintained below 1.5 kPa.

### Soluble carbohydrates and starch assays

In Experiment 1, whole shoots of the cultivars Paragon, Renan, and Récital were sampled in treatments HT.SD.320, HT.LD.170, HT.LD.280, and LT.LD.280(see Table 1) at Haun stage 3.5 at the end of the light and of dark periods for measurements of soluble sugars (glucose, fructose, and sucrose) and starch (soluble sugars and starch hereafter collectively referred to as carbohydrates). Six plants of each cultivar were sampled per treatment. Plants were immediately frozen in liquid nitrogen and stored at –80 °C prior to analysis. Plants were ground in liquid nitrogen using a mixer mill (MM 200, Retsch). Soluble sugars and starch were extracted and quantified by enzymatic assays following the procedure described by Hummel *et al.* (2010). Night consumption of carbohydrate (CC_night_, mg g^−1^ °Cd^−1^) was calculated as the difference in carbohydrate concentration between the measurements at the end of the day and the end of the night divided by the thermal time cumulated during the night.

### Modeling leaf appearance rate

Our newly developed LAR model (see Results) was implemented in the wheat phenology model described by He *et al.* (2012). This new phenology model was developed as an independent executable component in the BioMA software framework (http://www.biomamodelling.org). The BioMA component was integrated in the wheat model *SiriusQuality* (Martre *et al.*, 2006; Martre and Dambreville, 2018). The source code and the documentation of the BioMA component of the LAR model (Manceau and Martre, 2018) and the source code binaries of *SiriusQuality* and associated BioMA components (https://github.com/SiriusQuality/Release) are available under the MIT (X11) free and open-source software license.

 Our model of LAR (hereafter referred to as model M3) was compared with two other models. The first one (referred to as model M1) is a simple model where LAR expressed in thermal time unit is constant. The second one (referred to as model M2) is the LAR model used in the wheat model Sirius (Jamieson *et al.*, 2008) and described in detail by He *et al.* (2012). In model M2, leaf production follows a segmented linear model in thermal time. The first three leaves appear more rapidly than the next five, and LAR slows for the subsequent leaves independently of the total number of leaves produced. As a surrogate for the apex–air temperature correction for winter sowing (day of the year 1–90 for the Northern hemisphere), the phyllochron decreases linearly with the sowing date until reaching a minimum in mid-July for the Northern hemisphere (Jamieson *et al.*, 2008; He *et al.*, 2012). In the three LAR models, thermal time was calculated using Eqn 1 with the apex temperature assumed to be similar to soil temperature near its surface until Haun stage 4 and thereafter similar to the canopy temperature (Jamieson *et al.*, 1995). Soil and canopy temperatures were calculated from air temperature and the energy balances of soil surface and canopy, respectively (Jamieson *et al.*, 1995; Martre, 2013).

### Field experiments for model evaluation

Predictions from the three LAR models were compared against two field experiments with several sowing dates. The first one was the Hot Serial Cereal (HSC) experiment conducted in Maricopa (33°4ˈN, 111°58ˈW, 358 m elevation), AZ, USA, where the spring wheat cultivar Yecora Rojo was sown about every 6 weeks for 2 years (Wall *et al.*, 2011; White *et al.*, 2012). The data of the HSC experiment were obtained from Kimball *et al.* (2018). This experiment provides a very large range of temperature (average temperature between crop emergence and appearance of the flag-leaf = 9.6–22.3 °C) and photoperiod (10.1–13.9 h), with mean daily PTQ ranging from 1.2–3.8 mol m^−2^ °Cd^−1^. Only one year (height sowings) was used here as the results were very similar for the two years. The two summer sowings were not used as the crops died before they reached the flag-leaf ligule stage.

 The second experiment (hereafter referred to as NZ2020) was conducted over three consecutive winter growing seasons (2013–2014 to 2015–2016) in Canterbury, New Zealand, near Leeston (43°45ˈS, 172°15ˈE, 18 m elevation). Each year, the winter wheat cultivar Wakanui was sown at a density of 150 seeds m^−2^ at three (2013) or four dates (2014 and 2015) between late-February and late-April. Fertilizer, irrigation, insecticide, herbicide, and growth regulators were applied based on local practices. Four plots (replicates) were considered per treatment. Air temperature and relative humidity were recorded in a ventilated screen at 1.6 m height with a Campbell Scientific CS500 temperature and relative humidity probe. Solar radiation was measured in the field in 2013 and 2014 and correlated very closely with solar radiation measured at an automated weather station located at 65 km from the experimental site, so daily solar radiation data from the latter station were used for subsequent years. Following emergence, five plants were marked per plot and the number of ligules that appeared were recorded at 7–14 d intervals until flag-leaf ligule appearance. Plants produced 13–18 main-stem leaves and had a protracted tillering phase, so markers were moved up the stem to a recorded position following each measurement to keep an accurate record of the number of leaves that appeared.

### Estimation of LAR model parameters

LAR for models M1 and M2, and LAR_min_ for model M3 were estimated using the January 2009 sowing for the HSC experiment (Supplementary Fig. S1) and the second sowing date in 2014 and 2015 for the NZ200 experiment (Supplementary Fig. S2). In model M3, LAR_min_ and PTQ_hf_ were estimated using Eqn 3 (below) and the data we obtained from our experiments (see Results). *α* was estimated using the January 2009 sowing of the HSC experiment and the same value was used for both field experiments (Supplementary Table S2). The values of all parameters for the three models are given in Supplementary Table S2. Parameter values were estimated by minimizing the relative root-mean-squared relative error (RMSRE; see Supplementary Methods) for Haun stage >1.0 using a covariance matrix adaptation–evolutionary strategy (Hansen and Ostermeier, 2001) implemented in the *SiriusQuality* software.

### Data analysis and statistics

All data analyses and graphs were performed using the R statistical software version 3.4.1 (www.r-project.org). Differences in LAR_i_ between treatments and genotypes were determined using ANOVA. Genetic differences in the intercept and slope of the linear relationship between LAR_i_ and PTQ were analysed by reduced major-axis regression with the R package *smatr3* (Warton *et al.*, 2012). All statistical differences were judged at *P* < 0.05.

Depending on the range of PTQ, data for LAR_i_ versus PTQ were fitted using either a linear equation or a three-parameter asymptotic equation given as:

$$\begin{array}{l} LAR_{i}=LAR_{min}+\;\frac{(LAR_{max}-\;LAR_{min})\;\times \;PTQ}{PTQ_{hf}\;+\;PTQ} \end{array}$$ (3)

where, LAR_min_ (leaves °Cd^−1^) is the minimum LAR when PTQ equals zero, LAR_max_ (leaves °Cd^−1^) is the maximum LAR when PTQ tends to infinite, and PTQ_hf_ (mol PAR m^−2^ °Cd^−1^) is the PTQ at which LAR is half LAR_max_ + LAR_min_.

 Several statistics were calculated to assess the quality of the LAR models in the wheat model *SiriusQuality* (Supplementary Methods). Measured and simulated Haun stages were compared using the mean-squared error (MSE) and the root-mean-squared relative error (RMSRE). To get a better understanding of the model errors, the MSE was separated in non-unity slope (NU), squared bias (SB), and lack of correlation (LC; (Gauch *et al.*, 2003). To assess the model skill, we calculated the Nash–Sutcliffe modeling efficiency (EF; (Nash and Sutcliffe, 1970). To avoid confounding effects of leaf development and growth and auto-correlation in the data, MSE was calculated using the observed Haun stage value closest to 5 in all treatments. RMSRE was calculated to compare the models at different leaf stages, using all observed Haun stages >1.0.

## Results

### Leaf appearance rate depends on temperature, irradiance, photoperiod, and leaf stage

**Fig. 1.**
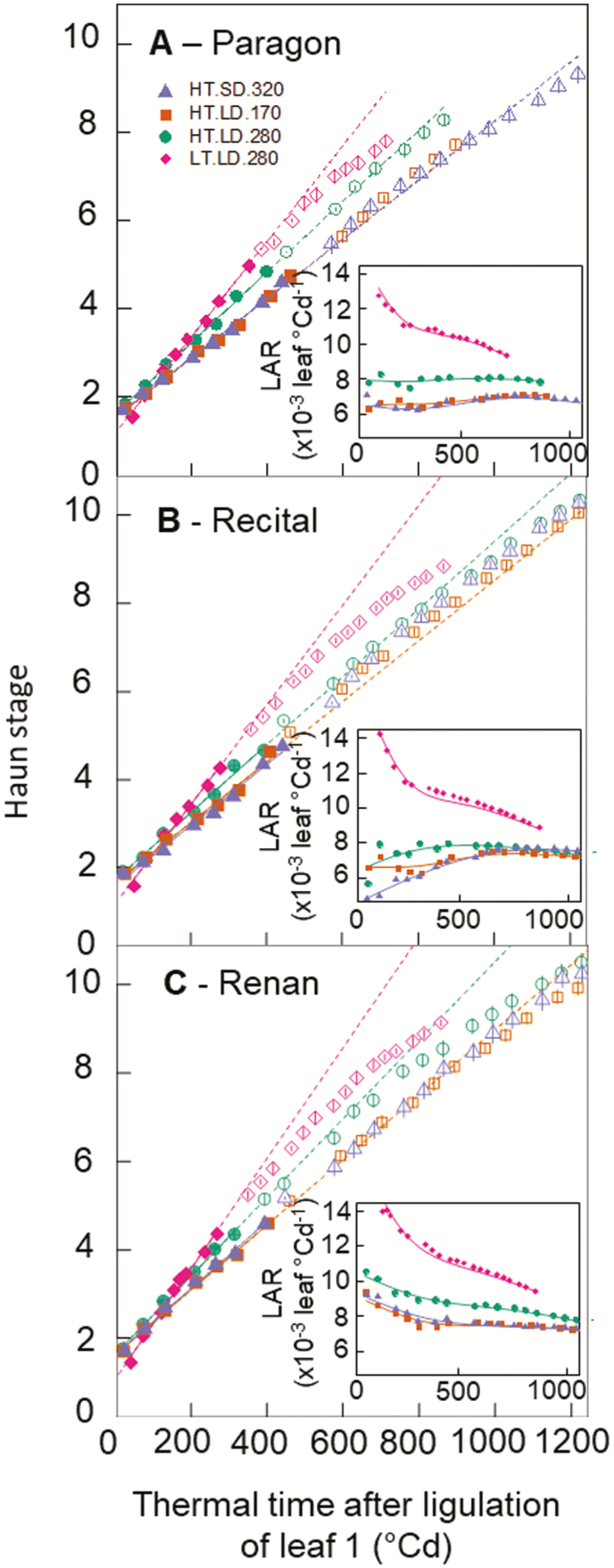
Relationships between Haun stage and thermal time after ligulation of leaf 1 for three wheat cultivars grown in controlled conditions with different temperatures, irradiances, and photoperiods. The photoperiod-insensitive spring wheat cultivar Paragon (A), the photoperiod-insensitive winter wheat cultivar Récital (B), and the photoperiod-sensitive winter wheat cultivar Renan (B) where grown in growth chambers with day/night conditions of air temperature 28/24 °C (high temperature, HT) or 18/14 °C (low temperature, LT), photoperiod 8 h (short days, SD) or 16 h (long days, LD), and PAR 170 µmol m^−2^ s^−1^ (170), 280 µmol m^−2^ s^−1^ (280), or 320 µmol m^−2^ s^−1^ 320). Treatments are detailed in Table 1. Lines are linear regressions calculated for Haun stage 1.5–5 (closed symbols; data for Huan stage >5 are shown as open symbols). The insets show the leaf appearance rate (LAR) versus thermal time after ligulation of leaf 1 and the lines are non-parametric spline curves fitted to the data. Thermal time was calculated using the apex temperature and Eqn 1. Data are means (±1SD) for *n*=6 independent replicates.

The dynamics of leaf appearance was first analysed for three contrasting cultivars (Fig. 1), Paragon (a photoperiod-insensitive spring wheat), Récital (a photoperiod-insensitive winter wheat), and Renan (a photoperiod-sensitive winter wheat), grown in four treatments with stable environmental conditions differing in temperature, photoperiod, and light intensity (Experiment 1; Table 1) in such a way that we could compare treatments differing in temperature only (LT.LD.280 versus HT.LD.280), irradiance only (HT.LD.280 versus HT.LD.170), or both irradiance and photoperiod but with a similar daily irradiance (HT.SD.170 versus HT.LD.170).

The initial leaf appearance rate (LAR_i_, calculated for leaves 1–5) differed significantly between treatments for the three cultivars (Fig. 1, Supplementary Table S3), although they had a similar response of LAR_i_ to the treatments (i.e. there were no significant treatment × cultivar interactions). The highest values of LAR_i_ were observed for the treatments with longer photoperiods and higher irradiance (LT.LD.280, HT.LD.280). For plants grown at high temperature, decreasing either the photoperiod (HT.SD.320) or the irradiance (HT.LD.280) decreased LAR_i_ (Fig. 1). Remarkably, changing both photoperiod and light intensity for a similar daily radiation and PTQ resulted in similar values of LAR (HT.SD.320 versus HT.LD.170).

In treatment LT.LD.280 (which showed the highest LAR_i_), LAR decreased with plant age for the three cultivars (insets in Fig. 1), including the winter cultivars (Fig. 1B, C), which stayed in the vegetative stage during the whole experiment. Therefore, the decrease of LAR with plant age in this treatment was related neither to floral transition nor to the development and formation of the spike. In the other treatments, LAR was either stable (for Paragon), increased (for Récital), or decreased (for Renan) with plant age. Overall, the LAR of all cultivar/treatment combinations converged towards the same value as the plants aged.

### Changes in leaf appearance rate with environmental conditions is a dynamic process

**Fig. 2.**
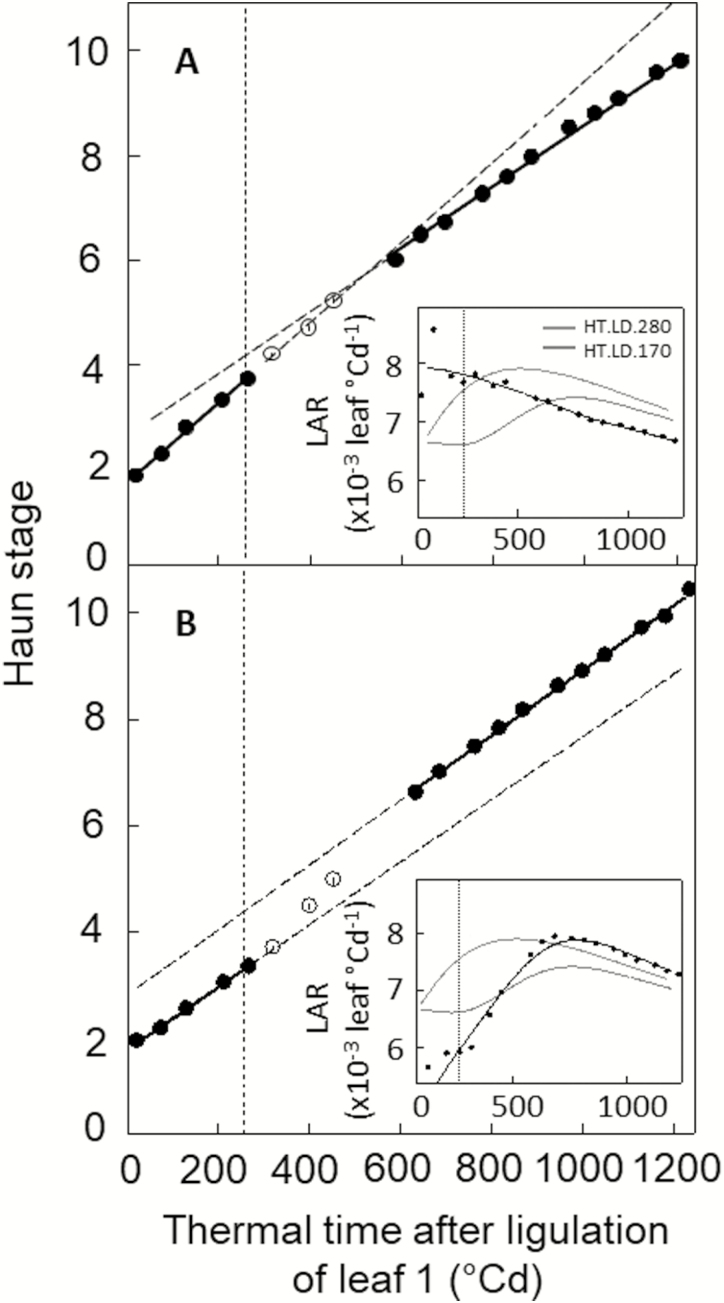
Relationships between Haun stage and thermal time after ligulation of leaf 1 for the photoperiod-insensitive winter wheat cultivar Récital grown in controlled conditions with different irradiance. The plants were grown from planting to 270 °Cd after ligulation of leaf 1 at 28/24 °C day/night air temperature and with a 16-h photoperiod and either (A) 280 µmol m^−2^ s^−1^ or (B) 170 µmol m^−2^ s^−1^ PAR (see Table 1). At 270 °Cd after ligulation of leaf 1, plants were transferred from low to high PAR (170→280) (A) or from high to low PAR (280→170) (B). Solid symbols are the data used to fit linear regressions before and after the change in irradiance. The insets show leaf appearance rate (LAR) versus thermal time after ligulation of leaf 1. Lines are non-parametric spline curves fitted to the data. Curves for treatments HT.LD.280 (green) and HT.LD.170 (pink) are from Fig. 1 and are shown for comparison. Thermal time was calculated using the apex temperature and Eqn 1. Data are means (±1SD) for *n*=6 independent replicates.

We analysed the dynamic changes in LAR with changes in irradiance. Plants from the two high-temperature plus long-photoperiod treatments were swapped between irradiance conditions at 270 °Cd after ligulation of leaf 1 (treatments 280→170 and 280→170). The environmental conditions before and after the irradiance swap were similar to treatments HT.LD.170 and HT.LD.280 in Fig. 1, in order to compare LARs at similar thermal time and to avoid confounding effects of plant age. Moreover, the winter wheat cultivar Récital was used to avoid confounding effects due to floral transition or spike development because it stayed in the vegetative stage during the whole experiment. The LAR of plants transferred from 280 µmol m^−2^ s^−1^ to 170 µmol m^−2^ s^−1^ PAR started to decrease about 250 °Cd (i.e. ~1.3 phyllochrons) after transfer to low irradiance, and the mean LAR after the transfer was 23% lower than before (Fig. 2A). The opposite behavior was observed when plants were transferred from 170 µmol m^−2^ s^−1^ to 280 µmol m^−2^ s^−1^ PAR, but LAR responded more rapidly to the change in irradiance (Fig. 2B). LAR increased within less than 140 °Cd (i.e. ~0.8 phyllochrons) after the transfer and the mean LAR was 12% higher than before.

### Leaf appearance rate is correlated with photothermal quotient, net daily photosynthesis, and carbohydrate turnover during the night

**Fig. 3.**
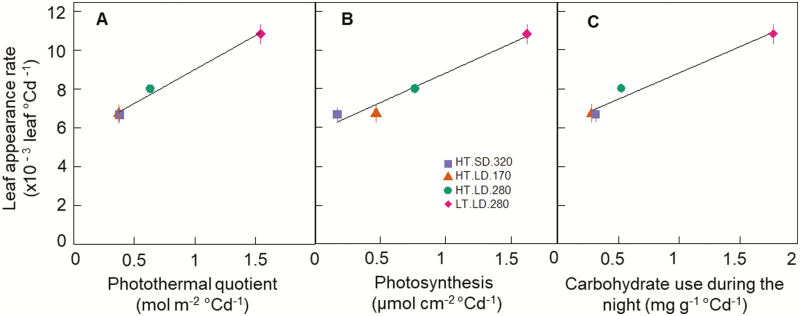
Relationships between initial leaf appearance rate (calculated for Haun stage ≤5) and photothermal quotient (A), net photosynthesis (B), and carbohydrate use during the night (C) for the photoperiod-insensitive spring wheat cv. Paragon grown in controlled conditions with different combinations of temperature, irradiance, and photoperiod (see Table 1). Thermal time was calculated using the apex temperature and Eqn 1. Treatments are as in Fig. 1. Data are means (±1SD) for *n*=6 independent replicates.

LAR was modified by temperature (even when expressed per unit thermal time), photoperiod, and instantaneous irradiance. To test whether these effects could be accounted for by the mean radiation per thermal-time unit, we calculated the photothermal quotient (PTQ) for all treatments in the three experiments, under ambient air CO_2_ concentration. The variation of LAR_i_ for cv. Paragon in Experiment 1 was well explained by a unique linear relationship linking LAR_i_ to PTQ independently of the cause of variation of PTQ (*r*^2^=0.965, *P*=0.018; Fig. 3A). A similar correlation was found between LAR_i_ and either daily net photosynthesis (*r*=0.982, *P*=0.0179; Fig. 3B) or carbohydrate consumption during the night (*r*=0.985, *P*=0.0147; Fig. 3C), supporting the hypothesis that LAR is at least partly limited by carbon availability in the plant.

**Fig. 4.**
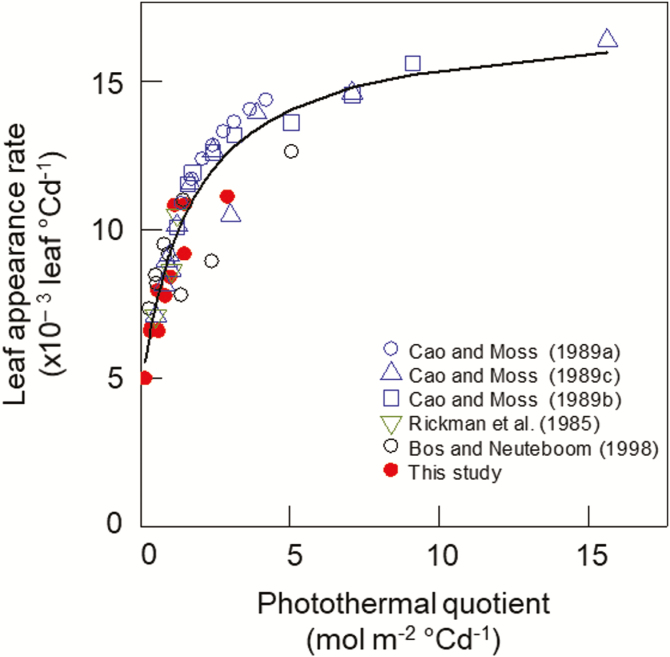
Relationships between leaf appearance rate and photothermal quotient for several wheat cultivars grown in controlled conditions with different combinations of temperature, irradiance, and photoperiod. Closed symbols are all treatments from Experiments 1–3 at ambient air CO_2_ concentration (Table 1). Open symbols are data from the literature (Supplementary Table S4). The curve is Eqn 3 fitted to all data points LAR=0.004996[±0.000731]+{(0.002364[±0.0007]−0.04996[±0.000731])×PTQ}/(1.9807[±0.5803]+PTQ).

The highest PTQ value tested in our experiments was 1.5 mol m^−2^ °Cd^−1^, while the range of PTQ sensed by plants in field conditions reached up to 4 mol m^−2^ °Cd^−1^ in our database of wheat field trials. The relationship between LAR and PTQ was therefore further tested on a larger range of PTQ values using data from the literature where the response of LAR to either temperature (Cao and Moss, 1989*b*, 1989*c*; Bos and Neuteboom, 1998), photoperiod (Cao and Moss, 1989*a*, 1989*c*), or irradiance (Rickman *et al.*, 1985; Bos and Neuteboom, 1998) was studied for plants grown in growth chambers or greenhouses (Supplementary Table S4). These data provide a very large range of variation of PTQ (up to 15 mol m^−2^ °Cd^−1^) and when our data and those from the literature were considered together the relationship between LAR and PTQ was described well by Eqn 3 (Fig. 4, Supplementary Table S5).

### Elevated CO_2_ increases leaf appearance rate at high temperatures

**Fig. 5.**
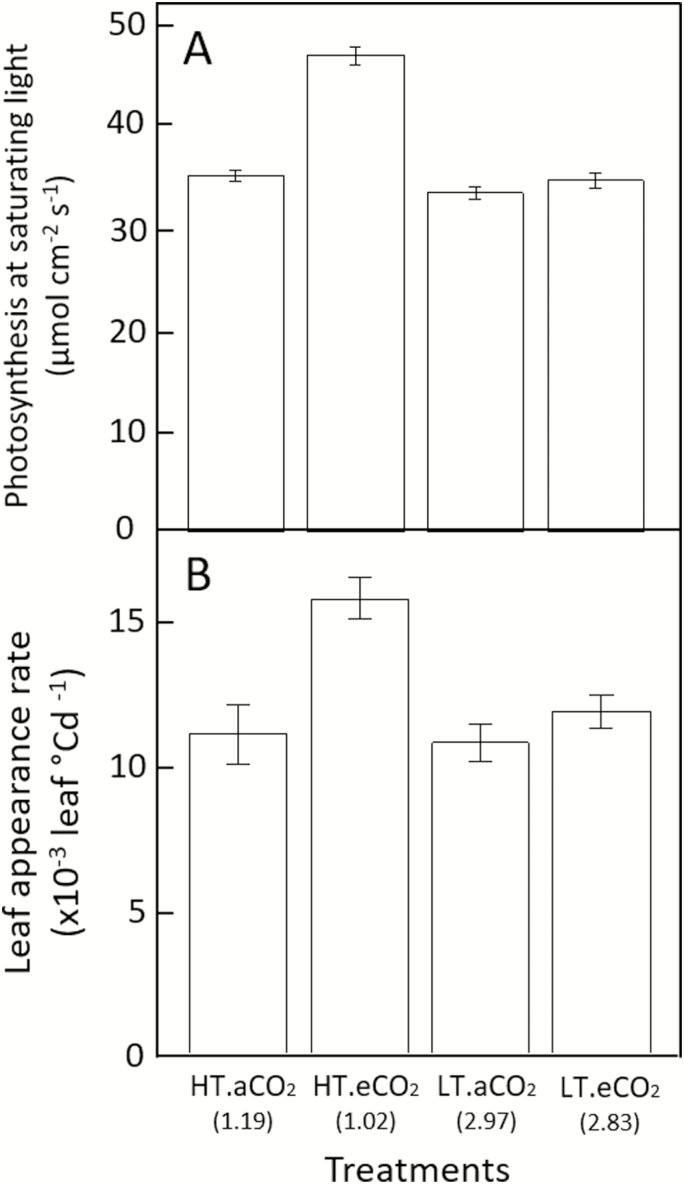
(A) Photosynthesis at saturating light and (B) initial leaf appearance rate (calculated for Haun stage ≤5) for the photoperiod-insensitive spring wheat cultivar Paragon grown in controlled environments with day/night conditions of air temperature of 18/14 °C (low temperature, LT) or 28/24 °C (high temperature, HT) and air CO_2_ concentration of 400 ppm (ambient, aCO_2_) or 800 ppm (elevated, eCO_2_). The value of the photothermal quotient (mol m^−2^ °Cd^−1^) is indicated below the treatment names. Details of the cultivars are given in Supplementary Table S1. Data are means (±1SD) for *n*=5 (A) or *n*=3 (B) independent replicates.

In order to strengthen our hypothesis of carbon limitation for LAR, we tested the effect of elevated air CO_2_ concentration on LAR_i_ (Experiment 2, Table 1). Plants of cv Paragon were grown under two temperature regimes (18/14 °C and 28/24 °C, day/night) and two atmospheric CO_2_ concentrations (400 ppm and 800 ppm). At 18/14 °C, photosynthesis under saturating light was not significantly different (*P*=0.064) between the two CO_2_ treatments (Fig. 5A), while at 28/24 °C it was 33.7% higher under elevated CO_2_ compared with ambient CO_2_ (*P*=7.3×10^−4^). Similarly, LAR_i_ was not significantly different between the CO_2_ treatments at 18/14 °C (*P*=0.019) but was 29.6% lower at 400 ppm than 800 ppm CO_2_ at 28/24 °C (*P*=1.54×10^−3^; Fig. 5B).

### Genetic variability of the response of leaf appearance rate to photothermal quotient

**Fig. 6.**
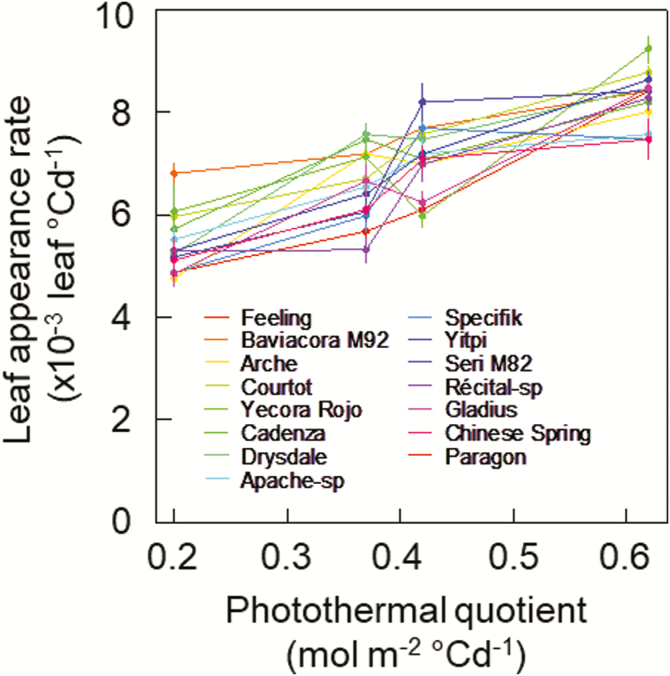
Relationships between initial leaf appearance rate (calculated for Haun stage ≤5) and photothermal quotient for 15 spring wheat cultivars grown in controlled conditions with different combinations of temperature and photoperiod (Table 1, Experiment 3). Details of the cultivars are given in Supplementary Table S1. Data are means (±1SD) for *n*=4 independent replicates.

We assessed the genetic variability of the response of LAR_i_ to PTQ for 15 spring wheat cultivars grown under two temperature regimes (18/14 °C and 28/24 °C, day/night) and two photoperiod treatments (8 h or 16 h) in factorial combination (Experiment 3, Table 1). The effect of PTQ on LAR_i_ was highly significant, while the effect of cultivar and the interaction between PTQ and cultivar were not significant (Fig. 6; Supplementary Table S6). The response of LAR_i_ to PTQ was analysed by linear regression (Supplementary Table S7). The slope of the LAR_i_–PTQ relationship was not significantly different among cultivars (*P*=0.77) and was on average 7.83 leaves m^2^ mol^−1^ PAR (95% confidence interval [CI] 6.90–8.90). However, the intercept of the relationship was significantly different among cultivars (*P* = 0.02) and ranged from 2.74×10^−3^ leaves °Cd^−1^ (CI 0.02–5.47) for cv. Feeling to 4.69×10^−3^ leaves °Cd^−1^ (CI 1.98–7.40) for cv. Cadenza.

### A model of carbon limitation of leaf appearance rate

We showed that differences in LAR_i_ due to temperature, light intensity, and photoperiod can be explained by a unique curvilinear relationship between LAR and PTQ (Fig. 4). PTQ reflects the balance between the incident irradiance available for growth and the potential growth of sinks driven by temperature. The demand for carbon for respiration scales with plant size and can be approximated by the green area index [GAI, m (leaf) m^−2^ (ground)]. The demand for carbohydrates for leaf growth increases between leaf 3 and terminal spikelet because of the regular formation and development of axillary tillers and associated roots (Kirby *et al.*, 1985; Abichou *et al.*, 2018). After terminal spikelet, growing leaves also compete for carbohydrates with fast-growing internodes and spikes. These changes in the source–sink balance during the plant growth cycle are at least partially compensated for by the increase in leaf area index. The decrease in LAR with ontogeny observed in our experiments (Fig. 1), as well as in many other studies (see Introduction), may reflect the decrease of the source–sink ratio with ontogeny.

We propose a simple model of LAR that summarizes the results above, in which (1) LAR depends on the supply-to-demand ratio for soluble carbohydrate, estimated by the ratio of intercepted light to thermal time; (2) the demand for soluble carbohydrate is proportional to plant size and this proportionality can be approximated by the green area index; (3) soluble carbohydrates in the plant provide a buffering capacity to fluctuating environments in the field; and (4) leaves are able to maintain a minimum rate of development. The model is given as:

$$\begin{array}{l} LAR=\frac{LAR_{min}+\left(\frac{\left[LAR_{max}-\;LAR_{min}\;\right]\times \left[\;\bar{I_{int}}\left(d\right)/\bar{T_{t}}\left(d\right)\right]}{PTQ_{hf}\;+\;\left[\bar{I_{int}}\left(d\right)/\bar{T_{t}}\left(d\right)\right]}\right)\;}{S_{C/GAI\;}\;\times \;GAI_{ef\;f}\left(d\right)\;} \end{array}$$ (4)

where, $\bar{I_{\text{int}}}\left(d\right)\;$[MJ PAR m^−2^ (ground)] is the cumulative PAR intercepted by the canopy during the period *d*, $\bar{T_{\text{t}}}\left(d\right)$ (°Cd) is the thermal time accumulated during the period *d*, ${\bar{\text{GAI}}}_{\text{eff}}$(*d*) [m (leaf) m^−2^ (ground)] is the mean green area fraction over the period *d*, *d* (°Cd) is the thermal time over which intercepted irradiance and thermal time are integrated, and *S*_C/GAI_ [m^2^ (ground) m^−2^ (leaf)] is an empirical parameter that scales carbon demand to GAI. In Eqn 4, LAR tends to infinite when GAI tends to 0. Therefore, a minimum value of ${\bar{\text{GAI}}}_{\text{eff}}$ was considered as the potential GAI when Haun stage = 3.5, just after the first tiller appears on the main stem. ${\bar{\text{GAI}}}_{\text{eff}}$ is given as:

$$\begin{array}{l} {\bar{GAI}}_{ef\;f}\left(d\right)=\left\{ \begin{array}{l} LN_{ef\;f}\times A_{L_{juv}}^{pot}\;\times PD,\;LN<LN_{ef\;f}\;\\ {\bar{GAI}}_{max}\left(d\right),\;LN\geq LN_{ef,f}\; \end{array}\right. \end{array}$$ (5)

where *A*^pot^_juv_ (cm^2^) is the potential surface area of juvenile leaves, as defined in the *SiriusQuality* leaf growth model (Martre and Dambreville, 2018), PD (plants m^−2^) is the plant density, LN (leaves main stem^−1^) is the number of emerged leaves on the main stem, LN_eff_ (leaves) is the number of main stem leaves above which the demand for respiration increases relative to sink formation, and ${\bar{GAI}}_{max}\left(d\right)$ is the maximum green area index fraction averaged over the period *d*, starting from emergence. The maximum value of $\bar{GAI}\left(d\right)$ is taken so that ${\bar{GAI}}_{ef\;f}$ does not decrease if the rate of senescence of the oldest leaves is higher than the expansion of the growing leaves.

In Eqn 4, environmental variables are averaged over several days to account for the buffering effect of stored soluble carbohydrates. The parameter *d* was set equal to 70 °Cd (Rickman *et al.*, 1985; Lattanzi *et al.*, 2005 ). The fraction of light intercepted by the crop during the period *d* is calculated from its exponential relationship with GAI (Monsi and Saeki, 2005).

### Prediction of leaf stage and leaf appearance rate for different sowing dates in the field

The three LAR models (M1, M2, and M3) were evaluated against two field experiments conducted in contrasting environments (HSC and NZ2020). In both experiments, LAR_i_ varied significantly with sowing dates. In the HSC experiment, LAR_i_ was constant and maximum for winter and spring sowings (between January and March, averaging 11.86×10^−3^ leaves °Cd^−1^) and decreased by 27% for the late-autumn sowing. In the NZ2020 experiment, crops were sown between late-summer and mid-autumn and LAR_i_ was constant and maximum for the first three sowings (averaging 10.45×10^−3^ leaves °Cd^−1^) but decreased on average by 25% for the latest sowing. The final number of leaves on the main stem was very different for the two experiments: 7.0–9.3 leaves main stem^−1^ for HSC and 17.7–12.8 leaves main stem^−1^ for NZ2020.

**Fig. 7.**
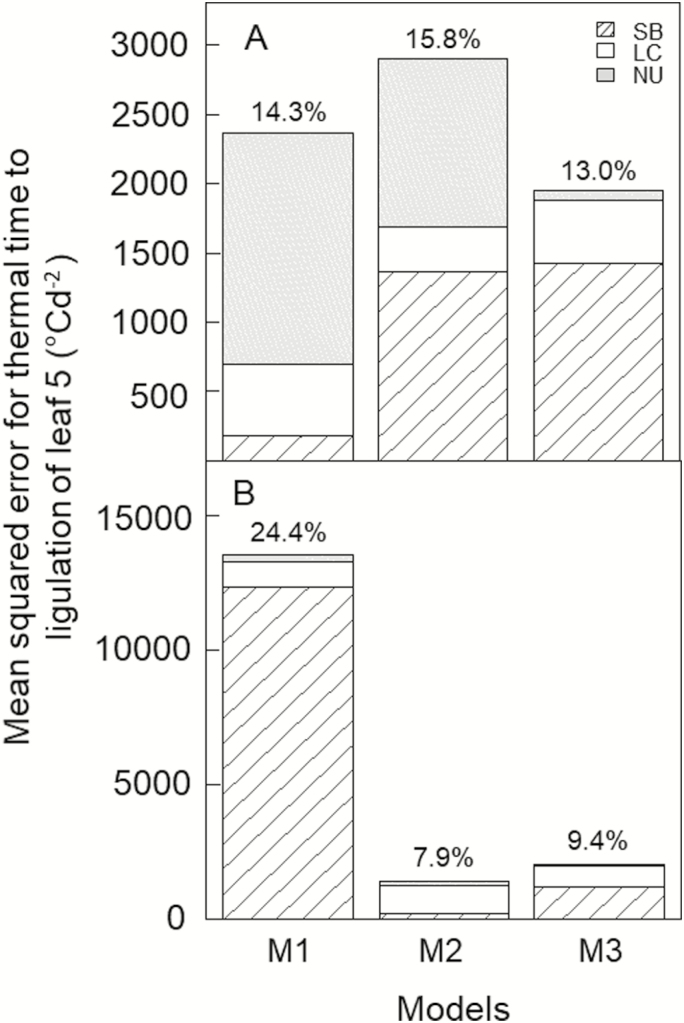
Mean-squared error (MSE) for thermal time to ligulation of leaf 5 (Haun stage 5) estimated using three alternative models of leaf appearance rate for (A) the photoperiod-insensitive spring wheat cultivar Yecora Rojo sown every ~6 weeks between March 2007 and January 2009 in the field at Maricopa, USA (HSC experiment), and (B) for the winter wheat cultivar Wakanui sown in the field in late-February, March, and April for three consecutive years at Leeston, New Zeland (NZ2020 experiment). MSE was decomposed in squared bias (SB), lack of correlation (LC), and non-unity slope (NU). MSE was calculated for the observed Haun stage closest to 5 to avoid confounding effects between leaf development and growth and autocorrelation in the data. Model M1, constant LAR; model M2, Sirius LAR model; model M3, LAR model developed in this study (see Methods).

In the HSC experiment, the error (MSE) of M3 for thermal time to Haun stage 5 was only 82% and 67% of that of M1 and M2, respectively (Fig. 7A). Lack of correlation (LC) contributed to 73% of the total error of M3, while the error of M1 was dominated by non-unity slope (NU) and that of M2 by squared bias (SB) and NU. In the NZ2020 experiment, the error for thermal time to Haun stage 5 was 85% lower for M3 than for M1 but was 41% higher for M3 than for M2 (Fig. 7). Squared bias and LC contributed nearly two-thirds and one-third of the total error of M3, respectively. Therefore, M3 had a much lower error than M1 for both data sets and outperformed M2 in Arizona, but in New Zealand both models had comparable and small errors (RMSRE<9.5%).

**Fig. 8.**
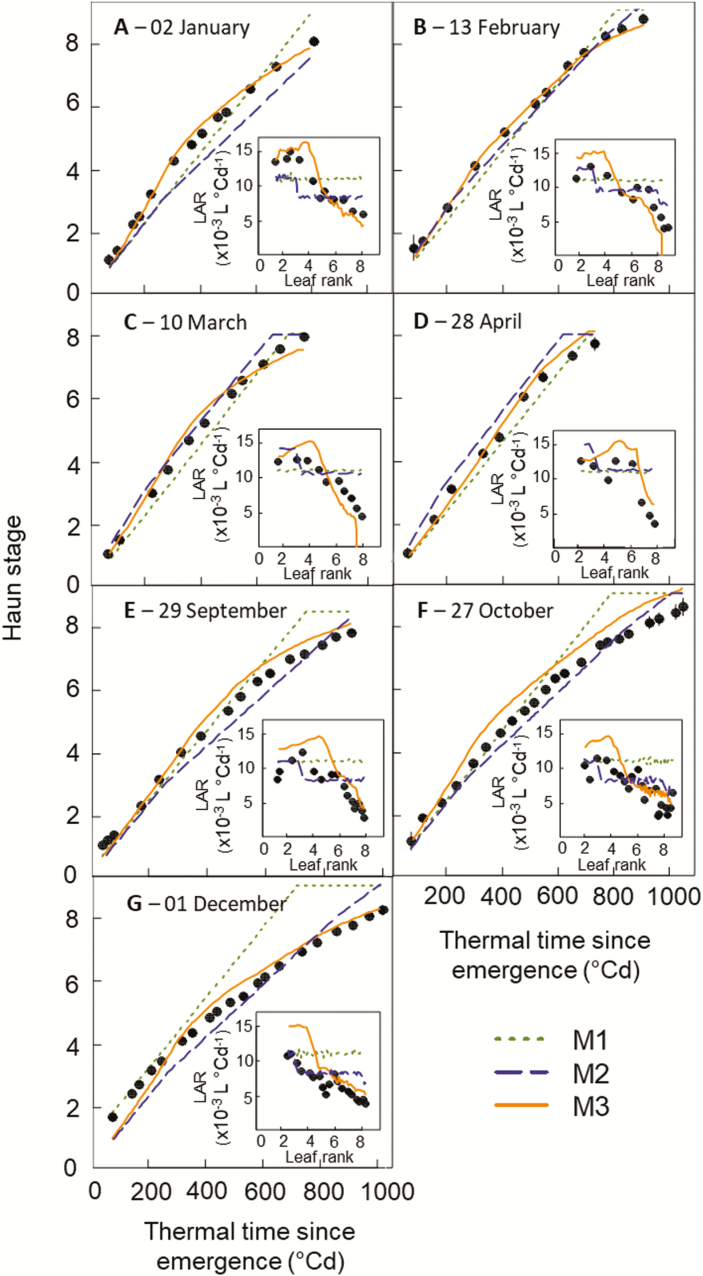
Relationships between observed (circles) and simulated (lines) Haun stage and thermal time after emergence for the spring wheat cultivar Yecora Rojo grown in field at Maricopa, Arizona, US (HSC experiment). Crops were sown every ~6 weeks between early January and early December 2008, as indicated in the figure. Lines are simulations obtained with the wheat model *SiriusQuality* and using either a constant phyllochron (model M1, dotted green lines), a segmented linear model in thermal time corrected for the sowing date (model M2, dashed blue lines), or Eqn 4 (model M3, solid orange lines). The insets show observed (circles) and simulated (lines) leaf appearance rate (LAR) versus leaf rank. Thermal time was calculated using the apex temperature and Eqn 1 for the observed data and canopy temperature for the simulated data (see Methods). Data are means ±1SD) for *n*=3 independent replicates.

**Fig. 9.**
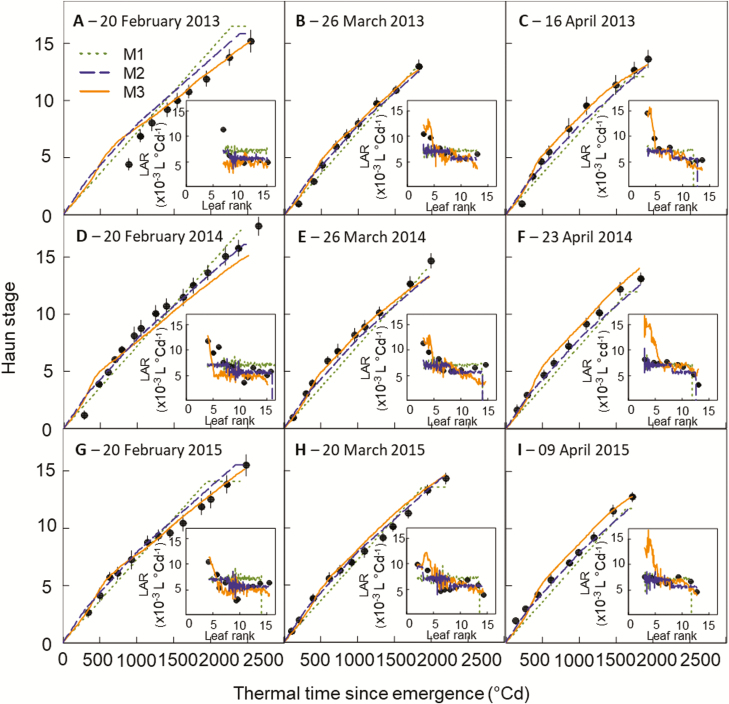
Relationships between observed (circles) and simulated (lines) Haun stage and thermal time after emergence for the spring wheat cultivar Wakanui grown in field at Leeston, New Zeland (NZ2020 experiment). Crops were sown from 2013 to 2015 in late-February, March, and April. Lines are simulations obtained with the wheat model *SiriusQuality* and using either a constant phyllochron (model M1, dotted green lines), a segmented linear model in thermal time corrected for the sowing date (model M2, dashed blue lines), or Eqn 4 (model M3, solid orange lines). The insets show observed (circles) and simulated (lines) leaf appearance rate (LAR) versus leaf rank. Thermal time was calculated using the canopy temperature and Eqn 1 for the observed data and canopy temperature for the simulated data (see Methods). Data are means (±1SD) for *n*=4 independent replicates.

In the HSC experiment, M3, which is based on biological hypotheses, provided a good simulation of the dynamics of leaf appearance and the observed changes of LAR with sowing date and plant ontogeny (Fig. 8). Compared with M1 and M2, the relative error (RMSRE) for Haun stage was reduced by 17% and 22%, respectively (Supplementary Table S8). M3 also simulated the dynamics of leaf appearance in the NZ2020 experiment reasonably well (Fig. 9) but the relative error for Haun stage was 10% higher for M3 than for M2 (Supplementary Table S8). Combining the two experiments, the overall RMSRE for Haun stage was 46% and 13% lower for M3 than for M1 and M2, respectively.

## Discussion

In this study, we investigated the effects of temperature, photoperiod, irradiance, CO_2_ concentration, and cultivars on wheat LAR. We showed that initial LAR (LAR_i_) changed significantly with all the factors studied excluding genotype (Figs 1, 5). We also showed that the response of LAR_i_ to environmental factors could be accounted for by the photothermal quotient (PTQ)(Fig. 4). LAR_i_ was also correlated with net photosynthesis and carbohydrate use at night (Fig. 3). Our results thus supported our hypothesis that LAR in wheat is carbon-limited. Based on our results, we developed and evaluated under field conditions a new model of LAR (Eqn 4) that accounts for both environmental and ontogenic changes in LAR (Figs 8, 9). The simulation results supported the modelling hypothesis that changes of LAR with ontogeny are due to changes in the carbon supply–demand ratio.

### Leaf appearance rate in wheat is carbon-driven

Relationships between temperature, irradiance, photoperiod, and LAR have been observed in a range of plant species, including cereals such as maize (Birch *et al.*, 1998), rice (Yin and Kropff, 1996), wheat and barley (Cao and Moss, 1989*c*; Volk and Bugbee, 1991; Bos and Neuteboom, 1998), and dicots such as quinoa (Bertero, 2001), lucerne (alfalfa, Brown *et al.*, 2005; Teixeira *et al.*, 2011), and lettuce (Kitaya *et al.*, 1998). However, no physiological explanation for the observed variations of LAR in relation to environmental factors has yet been proposed. Here, we found a unique relationship between LAR and PTQ for a large range of environmental conditions (Fig. 4). The fact that the photoperiod effect could be accounted for by a unique source–sink relationship for both photoperiod-insensitive and -sensitive spring and winter wheat cultivars was not expected. The treatments in our experiments allowed us to disentangle the effect of photoperiod *per se* and irradiance, and the results strongly suggested that the effect of photoperiod on LAR was mainly due to the increase of daily irradiance with longer photoperiods. An additional effect of photoperiod *per se* is not incompatible with our results, but this effect would be smaller compared to the effect of irradiance and would bring its own physiological determinisms and genetic variability. The correlation between LAR and net daily photosynthesis and carbon use during the night (Fig. 3), as well as the increase of LAR at elevated CO_2_ (Fig. 5), also supported the hypothesis that LAR in wheat is carbon-limited. In good agreement with our results,(McMaster *et al.* (1999) found that wheat plants grown under elevated CO_2_ (725 ppm) had values of LAR that were 10–15% higher than under ambient CO_2_ (360 ppm), and leaf photosynthesis and carbohydrate concentration were positively correlated with LAR.

Leaf appearance rate results from three processes: (1) cell division in the apical meristem of the expanding leaf primordium; (2) cell division of the intercalary meristem of the expanding leaf primordium; and (3) expansion of cells derived from the meristem in the leaf lamina and sheath. Christ and Körner (1995) showed that step-changes in CO_2_ concentration, and thus in carbon supply, have no effect on leaf elongation rate. This was most likely due to the fact that, in contrast to our current study, the leaves measured were initiated several plastochrons before the air CO_2_ concentration was increased. The effect of CO_2_ on wheat leaf growth acts mainly through an increased number of dividing cells at the base of expanding leaves, which is determined in the apical meristem before leaf appearance (Masle, 2000). The lack of correlation between soluble carbohydrate concentration in the elongation zone and leaf expansion rate after their emergence suggests that after leaves have emerged above the whorl of subtending leaves, their elongation rate is not limited by carbon availability (Kemp and Blacklow, 1980). This also agrees with studies showing that the control of leaf growth switches from a metabolic limitation to hydraulic and mechanical control during the course of leaf ontogeny (Pantin *et al.*, 2011).

### Changes of LAR with plant age reflect changes in the source–sink relationship

LAR decreases with plant age both in controlled conditions and in the field for wheat (Calderini *et al.*, 1996; Slafer and Rawson, 1997; Streck *et al.*, 2003; Ochagavía *et al.*, 2017) and also for other grass species such as sugarcane (Inman-Bamber, 1994) and tall fescue (Skinner and Nelson, 1995). But to date it has only been included in crop growth models through an empirical effect of leaf rank on LAR (Jamieson *et al.*, 1995) or through an effect of the distance from the meristem to the whorl (Streck *et al.*, 2003). In our results, the decrease of LAR with plant age depended on environmental conditions (Figs 1, 2), which was incompatible with a unique relationship linking LAR and Haun Stage (Streck *et al.*, 2003).

As LAR depends on plant carbon availability, it is tempting to hypothesize that the decrease of LAR with plant age is associated with changes in plant source–sink balance and with a lower availability of carbon. As the wheat plant develops, the formation and development of new tillers increases the demand for carbon, and after the terminal spikelet stage expanding leaves also compete for carbon with the growing internodes and spikes. In tall fescue, LAR decreases rapidly after the appearance of leaf 7, and this can be suppressed if new tillers are trimmed (Skinner and Nelson, 1995). In that study, the decrease in LAR with leaf number was due both to an increase of the duration of the leaf elongation through the whorl of subtending leaves and to a decrease of the interval between the initiation of successive leaves, and both may be due to the slowing down of leaf elongation rate (Skinner and Nelson, 1995). Slafer and Rawson (1997) reported that LAR after leaf 6 is more sensitive to photoperiod than that of leaves appearing before. This is in good agreement with a carbon-limitation of LAR, and the results of (Skinner and Nelson, 1995) in tall fescue.

### Consideration of the carbon-limitation of leaf appearance improves the prediction of leaf stages in the field

Many crop growth models calculate leaf appearance assuming a constant LAR and do not consider the effects of photoperiod, light intensity, or plant age. Where there have been attempts to model the response of LAR to photoperiod, these have been empirical models (Jamieson *et al.*, 2008; Abichou *et al.*, 2018; Brown *et al.*, 2018) and they are probably limited in the range of environmental conditions in which they can be used. Moreover, these models have a large number of parameters. Here, we present an ecophysiological model (Eqn 4) that can easily be integrated into crop growth models and has 40% fewer parameters than the Sirius LAR model. Our model was able to simulate the changes in LAR with both sowing date and plant age in two contrasting environments.

Several crop growth models do not use leaf stages and leaf number to simulate heading or anthesis date, but instead use a more empirical approach based on the thermal-time requirement between phenological phases and modifications of thermal time by vernalization and photoperiod (e.g. Ritchie, 1991; Stöckle *et al.*, 2003; Brisson *et al.*, 2009). One of the reasons such phenology models are used in crop growth models is that the error in leaf-stage prediction with the leaf-number approach may lead to large errors in the prediction of anthesis date. Although these two types of approaches may provide very similar results (Jamieson *et al.*, 2007), models based on leaf number allow for separation of the effect of temperature on development and vernalization (Allard *et al.*, 2012) and better represent biological processes, and thus can more directly be related to physiological processes or even genes, for instance those controlling flowering time (Brown *et al.*, 2013; Sanna *et al.*, 2014). A phenology model based on leaf number also allows the linking of phenology with leaf growth (Lawless *et al.*, 2005; Martre and Dambreville, 2018). The improvement of leaf-stage modeling provided by our model is thus an important step to improve models based on leaf number and to introduce more physiological insights into crop growth models.

## Supplementary data

Supplementary data are available at *JXB* online.

Methods. Details of statistics used for model evaluation.

Table S1. Details of the cultivars used in this study.

Table S2. Non-varietal and varietal parameters of the Sirius phenology sub-model.

Table S3. ANOVA for the responses of LAR_i_ to PTQ and cultivar shown in Fig. 1.

Table S4. Environmental conditions and LAR_i_ taken from the literature and shown in Fig. 4.

Table S5. ANOVA for the responses of LAR_i_ to temperature, CO_2_, and cultivar shown in Fig. 5.

Table S6. ANOVA for the responses of LAR_i_ to PTQ and cultivar shown in Fig. 6.

Table S7. Summary statistics of the linear regression analysis of LAR_i_ versus PTQ for 15 spring wheat cultivars.

Table S8. Model errors and skills for leaf stage.

Fig. S1. Relationship between observed and simulated Haun stage and thermal time since emergence for the HSC experiment.

Fig. S2. Relationship between observed and simulated Haun stage and thermal time since emergence for the NZ2020 experiment

Fig. S3. Net photosynthesis and carbohydrate concentration responses for the wheat cv Paragon grown under different conditions of temperature, irradiance, and photoperiod.

Supplementary Material
