# Supplementary material for: Experimental and modeling evidence of carbon limitation of leaf appearance rate for spring and winter wheat
Source: J Exp Bot. 2019 Feb 20;70(9):2449–62. doi: 10.1093/jxb/erz012 (PMC6487592; doi:10.1093/jxb/erz012)
Supplement: Supplementary Material [file erz012_suppl_supplementary_material.pdf]

Supplementary data

Experimental and modeling evidences of carbon-limited leaf appearance rate for spring and winter wheat

Maeva Baumont, Boris Parent, Hamish Brown, Steven M. Driever, Bertrand Muller, Pierre Martre

Content

Supplementary Methods

Table S1. Details of the cultivars used in this study.

Table S2. Parameters of the LAR models compared in this study.

Table S3. Analysis of variance for the response of LARi to PTQ and cultivar.

Table S4. Environmental conditions and LARi from the literature.

Table S5. Analysis of variance for the response of initial leaf appearance rate to temperature, CO_2 and cultivar.

Table S6. Analysis of variance for the response of LARi to PTQ and cultivar shown in Figure 5

Table S7. Summary statistics of the linear regression analysis of LARi versus PTQ for 15 spring wheat cultivars.

Table S8. Model errors for leaf stage.

Figure S1. Relationship between observed and simulated Haun stage and thermal time since emergence for January 2009 HSC sowing.

Figure S2. Relationship between observed and simulated Haun stage and thermal time since emergence for early March 2014 and 2015 NZ sowing.

Figure S3. Photosynthesis at saturating light for the spring wheat cv. Paragon grown in controlled conditions at two temperature regimes and two air CO_2 concentration.

Supplementary Methods

Statistics for model evaluation

To assess the quality of the LAR models in the wheat model *SiriusQuality* measured (y_i) and simulated ($\widehat{y_i}$) thermal time at Haun stage 5 were compared using the mean squared error (MSE):

$$\mathrm{MSE} = \frac{1}{n}\sum_{i=1}^{n}(y_i - \widehat{y_i})^2 \tag{S1}$$

The relative root mean squared error (RRMSE) was calculated as:

$$\mathrm{RRMSE} = 100 \times \frac{\sqrt{\frac{1}{n}\sum_{i=1}^{n}(y_i - \widehat{y_i})^2}}{\overline{y}} \tag{S1}$$

where $\overline{y}$ is the average of the observed values. To get a better understanding of the model errors we decomposed the MSE in non-unity slope (NU), squared bias (SB) and lack of correlation (LC; Gauch et al., 2003):

$$\mathrm{NU} = (1 - b^2)\left(\frac{\left(\sum_{i=1}^{n} y_i\right)^2}{n}\right) \tag{S2}$$

$$SB = \left(\overline{y_i} - \overline{\widehat{y_i}}\right)^2 \tag{S3}$$

$$\mathrm{LC} = (1 - r^2)\left(\frac{\left(\sum_{i=1}^{n} \widehat{y_i}\right)^2}{n}\right) \tag{S4}$$

where, b is the slope of the regression of $\widehat{y_i}$ on $y_i$and r^2 is the coefficient of correlation.

The three components of the MSE, which add up to give MSE, represent different aspects of the overall deviation of the model simulations and have simple geometrical interpretations. NU reflects the rotation, SB the translation, and LC the scattering (random error) around the 1:1 line. This analysis was used in complement of the classical least square linear regression. To avoid confounding effects of leaf development and growth, and auto-correlation in the data and MSE analyses were performed using the observed Haun stage value closest to five in all treatments.

The root mean squared relative error (RMSRE) was calculated to compare the models at different leaf stages:

$$\mathrm{RMSRE} = \sqrt{\frac{1}{n}\sum_{i=1}^{n}\left(\frac{y_i - \widehat{y_i}}{y_i}\right)^2} \tag{S6}$$

RMSRE was calculated using all observed Haun stage values > 1.0.

Table S1. Name, country of origin, registration year, growth habit, and photoperiod sensitivity and genotype at the Vrn and Ppd loci of the 17 wheat genotypes (herein referred as cultivars) used in this study.

Name	Status	Country of origin[*]	Registration year[*]	Growth habit[*]	Vernalization genes[£]				Photoperiod sensitivity	Photoperiod sensitivity genes[#]		
					Vrn-A1[$]	Vrn-B1[$]	Vrn-D1[$]	VRN-B3[¥]		Ppd-A1[$]	Ppd-B1[$]	Ppd-D1[$]
Apache-sp[1]	Near isogenic line	France	NA	Spring	**5**	1	1	NA	Insensitive	NA	b	***a***
Arche[2]	Cultivar	France	1989	Spring	**5**	1	1	2	Sensitive	b	b	b
Baviacora M92	Cultivar	Mexico	1992	Spring	v	v	***a***	NA	Insensitive	b	***a***	***a***
Cadenza[2]	Cultivar	England	1993	Spring	***a***	***2***	1	2	Sensitive	c	b	b
Chinese spring[2]	Landrace	China	NA	Spring	**2**	1	2	2	Insensitive	b	a	b
Courtot[2]	Cultivar	France	1974	Spring	**5**	***2***	1	2	Sensitive	NA	b	b
Drysdale[3]	Cultivar	Australia	2001	Spring	v	***a***	***a***	NA	Insensitive	NA	***a***	***a***
Feeling	Cultivar	France	2015	Spring	NA	NA	NA	NA	NA	NA	NA	NA
Gladius[3]	Cultivar	Australia	2007	Spring	***a***	***a***	v	NA	Sensitive	NA	***a***	b
Paragon	Cultivar	England	1998	Spring	***a***	NA	NA	NA	Sensitive	b	b	b
Récital[2]	Cultivar	France	1986	Winter	2	1	1	2	Insensitive	a	***a***	***a***
Récital-sp[1]	Near isogenic line	France	NA	Spring	***a***	***a***	***a***	NA	Insensitive	NA	***a***	***a***
Renan	Cultivar	France	1989	Winter	2	1	1	2	Sensitive	b	b	c
Seri M82[3]	Cultivar	Mexico	1982	Spring	v	***a***	***a***	NA	Insensitive	a	***a***	***a***
Specifik	Cultivar	France	2010	Spring	NA	**2**	1	NA	Sensible	NA	NA	b
Yecora Rojo	Cultivar	USA	1975	Spring	2-3	**2**	1	NA	Insensitive	b	b	**a**
Yitpi[3]	Cultivar	Australia	2000	Spring	***a***	***a***	v	NA	Insensitive	NA	**a**	d

[1] Isogenic line of the winter wheat cultivar Récital and Apache introgressed with the Vrn-A1pr-5 spring allele (Rousset el al, 2011) of Arche.
[2] Vernalization and photoperiod genes information given by Rousset et al. (2011).
[3] Vernalization and photoperiod genes information given by Zheng et al. (2011).
[*] Source: http://www.wheatpedigree.net/
[$] The Vrn spring alleles and Ppd photoperiod insensitive alleles are in bold italic type.
[£] Mutant (spring type) is semi-dominant. Mutation of one homeologous gene is sufficient to give a spring growth habit.
[¥] Mutant (spring type) is semi-dominant.
[#] Relative potency of the reduction in flowering time for the photoperiod insensitive variants: Ppd-D1a=Ppd-A1a>Ppd-B1a.

Table S2. Name, definition, value and unit of the parameters of the leaf appearance rate (LAR) models compared in this study. Model M1, constant LAR; model M2, segmented linear model in thermal time corrected for the sowing date (He *et al*., 2012); model M3, LAR model proposed in this study (Eq. 4).

Name	Definition	Value		Unit
		Yecora Rojo	Wakanui	
**Model M1**				
LAR	Leaf appearance rate	0.0111	0.00714	leaf °Cd^{-1}
**Model M2**				
LAR	Leaf appearance rate	0.00833	0.00714	leaf °Cd^{-1}
L_{incr}	Haun stage above which LAR is increased by P_{incr}	8	8	leaf
L_{decr}	Haun stage up to which LAR is decreased by P_{decr}	3	3	leaf
P_{incr}	Factor increasing LAR for leaf number higher than or equal to L_{incr}	1.25	1.25	dimensionless
P_{decr}	Factor decreasing LAR for leaf number less than L_{decr}	0.75	0.75	dimensionless
R_{P}	Rate of decrease of LAR for winter sowing	0.003	0.003	°Cd d^{-1}
$SD_{\mathrm{W/S}}$	Sowing date for which LAR is maximum	90	90	day of the year
$SD_{S/A}^{nh}$	Sowing date for which LAR is minimum in the northern hemisphere	200	-	day of the year
$SD_{S/A}^{sh}$	Sowing date for which LAR is minimum in the southern hemisphere	-	151	day of the year
**Model M3**				
α	Scaling coefficient of carbon demand to green area index	1.26	1.26	m^{2} (ground) m^{-2} (leaf)
d	Thermal time over which intercepted irradiance and thermal time are integrated	70	70	°Cd
$\mathrm{LAR}_{\mathrm{min}}$	Leaf appearance rate for photothermal quotient equals zero	0.0138	0.005	leaf °Cd^{-1}
$\mathrm{LAR}_{\mathrm{max}}$	Maximum leaf appearance rate when photothermal quotient tends to infinite	0.0264	0.0264	leaf °Cd^{-1}
$\mathrm{PTQ}_{\mathrm{hf}}$	Photothermal quotient when leaf appearance rate is half $\mathrm{LAR}_{\mathrm{max}} + \mathrm{LAR}_{\mathrm{min}}$	0.46	0.46	MJ (PAR) m^{-2} °Cd^{-1}

Table S3. Analysis of variance for the response of initial leaf appearance rate to environmental treatment (E) and genotype (G) shown in Figure 1.					
Effect	**Degree of freedom**	**Sum of squares**	**Mean squares**	**F-value**	***P*-value**
E	1	4.12 x10^{-5}	4.12 x10^{-5}	177.38	9.65 x10^{-7}
G	1	9.96 x10^{-7}	9.96 x10^{-7}	4.29	0.072
G x E	1	2.11 x10^{-7}	2.11 x10^{-7}	0.91	0.368
Residuals	8	12.360	0.281	-	-

Table S4. Environmental conditions and initial leaf appearance rate (LARi) from the literature sown in Figure 4. Average daily thermal time was calculated using Eq. (1).								
**Reference / Cultivar**	**Set point day/night air temperature**	**Set point PAR**	**Photoperiod**	**Set point day/night air VPD**	**Average daily PAR**	**Average daily thermal time**	**PTQ**	**LARi**
	**(°C)**	**(µmol m^{-2} s^{-1})**	**(h)**	**(kPa) [a]**	**(mol m^{-2} d^{-1})**	**(°C)**	**(mol m^{-2} °Cd)**	**(x 10^{-3} leaf $°Cd^{-1}$)**
**Cao and Moss (1989a) – Growth chamber, photoperiod**								
Stephens	15/15	400	8	NA	11.52	8.18	1.41	10.87
	15/15	400	10	NA	14.40	8.18	1.76	11.71
	15/15	400	12	NA	17.28	8.18	2.11	12.38
	15/15	400	14	NA	20.16	8.18	2.46	12.85
	15/15	400	16	NA	23.04	8.18	2.82	13.28
	15/15	400	18	NA	25.92	8.18	3.17	13.60
	15/15	400	21	NA	30.24	8.18	3.70	14.04
	15/15	400	24	NA	34.56	8.18	4.22	14.36
**Cao and Moss (1989c) – Growth chamber, temperature**								
Stephens	7.5/7.5	400	14	NA	20.16	1.29	15.63	16.38
	10/10	400	14	NA	20.16	2.84	7.10	14.61
	12.5/12.5	400	14	NA	20.16	5.14	3.92	13.91
	15/15	400	14	NA	20.16	8.18	2.46	12.64
	17.5/17.5	400	14	NA	20.16	11.88	1.70	11.54
	20/20	400	14	NA	20.16	16.04	1.26	10.15
	22.5/22.5	400	14	NA	20.16	20.34	0.99	9.15
	25/25	400	14	NA	20.16	24.35	0.83	8.15
**Cao and Moss (1989b) – Growth chamber, photoperiod x temperature interactions**								
Stephens	20/20	400	6	NA	8.64	16.04	0.54	7.1
	15/15	400	6	NA	8.64	8.18	1.06	8.63
	10/10	400	6	NA	8.64	2.84	3.04	10.49
	20/20	400	10	NA	14.40	16.04	0.90	8.95
**Table S4.** Continued.								

Stephens	15/15	400	10	NA	14.40	8.18	1.76	11.91
	10/10	400	10	NA	14.40	2.84	5.07	13.60
	20/20	400	14	NA	20.16	16.04	1.26	10.08
	15/15	400	14	NA	20.16	8.18	2.46	12.61
	10/10	400	14	NA	20.16	2.84	7.10	14.52
	20/20	400	18	NA	25.92	16.04	1.62	11.53
	15/15	400	18	NA	25.92	8.18	3.17	13.19
	10/10	400	18	NA	25.92	2.84	9.13	15.61
**Rickman et al., (1985) – Green house, irradiance**								
Stephens	17/17	500	12	0.82 / 0.82	10.8	11.10	1.95	7.10
	17/17	275	12	0.82 / 0.82	5.94	11.10	1.07	8.63
	17/17	140	12	0.82 / 0.82	3.02	11.10	0.54	10.49
**Bos and Neuteboom (1998) – Growth chamber, irradiance x temperature interactions**								
Minaret	18/13	111	14	0.61 / 0.45	5.59	9.76	0.57	8.20
	18/13	191	14	0.61 / 0.45	9.63	9.76	0.99	9.17
	18/13	286	14	0.61 / 0.45	14.41	9.76	1.48	10.99
	23/18	111	14	0.84 / 0.61	5.59	17.64	0.32	7.35
	23/18	191	14	0.84 / 0.61	9.63	17.64	0.55	8.47
	23/18	286	14	0.84 / 0.61	14.41	17.64	0.82	9.52
	13/8	111	14	0.45 / 0.32	5.59	3.96	1.41	7.81
	13/8	191	14	0.45 / 0.32	9.63	3.96	2.43	8.93
	13/8	286	14	0.45 / 0.32	20.16	3.96	5.09	12.61

[a] NA, not available.

Table S5. Analysis of variance for the response of initial leaf appearance rate to temperature (T), air CO_2 concentration (CO_2) and genotype (G) effects shown in Figure 4.					
Effect	**Degree of freedom**	**Sum of squares**	**Mean squares**	***F*-value**	***P*-value**
CO2	1	1.11 x10^{-04}	1.11 x10^{-04}	246.7	< 2.00 x10^{-16}
T	1	1.41 x10^{-05}	1.41 x10^{-05}	31.4	1.14 x10^{-06}
G	2	6.17 x10^{-05}	3.08 x10^{-05}	68.7	1.53 x10^{-14}
CO_2 x T	1	2.73 x10^{-05}	2.73 x10^{-05}	60.9	5.80 x10^{-10}
CO_2 x G	2	2.35 x10^{-05}	1.17 x10^{-05}	26.2	2.59 x10^{-08}
T x G	2	1.27 x10^{-05}	6.37 x10^{-06}	14.2	1.59 x10^{-05}
CO_2 x T x G	2	3.16 x10^{-06}	1.58 x10^{-06}	3.5	0.0378
Residuals	46	2.07 x10^{-05}	4.50 x10^{-07}	-	-

Table S6. Analysis of variance for the response of initial leaf appearance rate to photothermal quotient (PTQ) and genotype (G) shown in Figure 5.					
Effect	**Degree of freedom**	**Sum of squares**	**Mean squares**	**F-value**	***P*-value**
PTQ	1	63.24	63.24	225.14	$< 2.00 \times 10^{-16}$
G	14	7.05	0.50	1.49	0.17
PTQ x G	14	2.21	0.16	0.47	0.93
Residuals	30	10.15	0.33	-	-

Table S7. Summary statistics of the linear regression analysis of initial leaf appearance rate versus photothermal quotient for 15 spring wheat cultivars grown in controlled conditions with different temperature and photoperiod (Experience 3, Table 1). CI, confidence intervals.

Cultivar	r^2	*P*-value	**Slope (leaf m^2 mol^{-1} PAR)**			**Intercept (× 10^{-3} leaf °Cd^{-1})**	
			**Estimated**	**95% CI**		**Estimated**	**95% CI**
Apache-sp	0.902	0.050	5.13	2.20 - 12.10		4.62	2.49 - 6.74
Arche	0.852	0.076	8.02	2.96 - 21.69		3.51	-0.52 - 7.54
Baviacora M92	0.960	0.020	3.99	2.24 - 7.09		5.92	4.87 - 6.95
Cadenza	0.881	0.061	6.02	2.41 - 15.01		4.69	1.98 - 7.40
Chinese Spring	0.879	0.063	6.07	2.41 - 15.29		4.00	1.22 - 2.77
Courtot	0.965	0.017	7.00	4.09 - 11.99		4.43	2.73 - 6.13
Drysdale	0.872	0.066	7.96	3.11 - 20.42		3.98	0.26 - 7.71
Feeling	0.943	0.029	8.74	4.46 - 17.14		2.74	0.02 - 5.47
Gladius	0.946	0.027	8.63	4.46 - 16.67		3.09	0.46 - 5.71
Paragon	0.994	0.003	6.71	5.32 - 8.45		4.07	3.39 - 4.74
Recital-sp	0.819	0.094	8.32	2.84 - 24.33		3.12	-1.51 - 7.74
Seri M82	0.772	0.121	9.19	2.86 - 29.54		3.26	-2.49 - 9.01
Specifik	0.704	0.161	7.64	2.13 - 27.42		3.43	-2.03 - 8.89
Yecora Rojo	0.692	0.167	8.73	2.39 - 31.87		3.58	-2.78 - 9.95
Yitpi	0.987	0.006	8.09	5.77 - 11.34		3.62	2.42 - 4.81
Overall	0.765	$< 2 \times 10^{-16}$	6.86	6.90 - 8.90		4.07	3.24 - 4.11

Table S8. Root mean squared relative error (RMSRE) calculated for Haun stage > 1.0 for the HSC and NZ2020 experiments, and for both experiments together (overall). Model M1, constant LAR; model M2, Sirius LAR model; model M3, LAR model proposed in this study.

Model	RMSRE (%)		
	**HSC**	**NZ2020**	**Overall**
**M1**	15.04	25.32	20.58
**M2**	15.77	8.39	12.80
**M3**	12.49	9.26	11.08

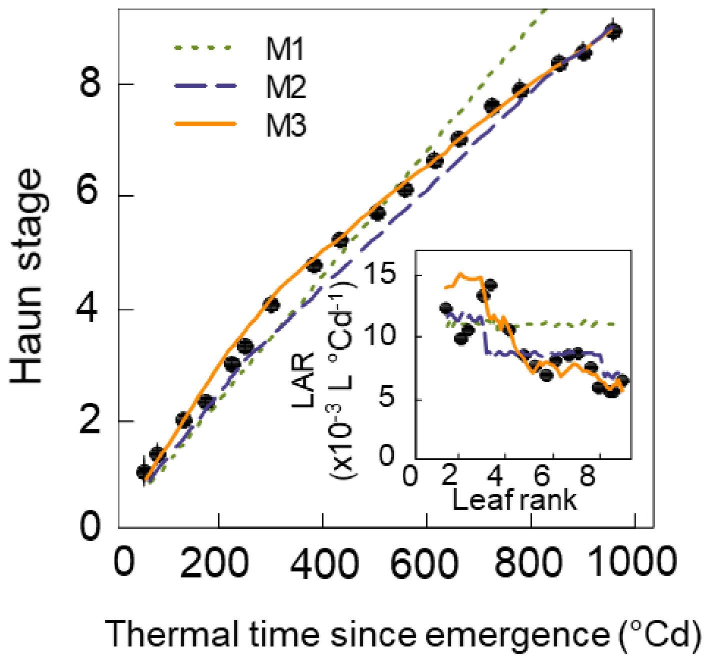

Figure S1. Relationship between observed (close circle) and simulated (lines) Haun stage and thermal time after emergence for the spring wheat cultivar Yecora Rojo sown in the field at Maricopa, Arizona US on 12 January 2009. Lines are simulations obtained with the wheat model *SiriusQuality* and using either a constant phyllochron (model M1, dotted green lines), a segmented linear model in thermal time corrected for the sowing date (model M2, dashed blue lines) or our new model (model M3, solid orange lines). Inset shows observed (closed circles) and simulated (lines) leaf appearance rate versus leaf rank. The thermal time was calculated using apex temperature and Eq. (1) for the observation and using simulated near-surface soil or canopy temperature and Eq. (1) for the simulated data (see Material and Methods). Data are mean ± 1 s.d. for n = 3 independent replicates.

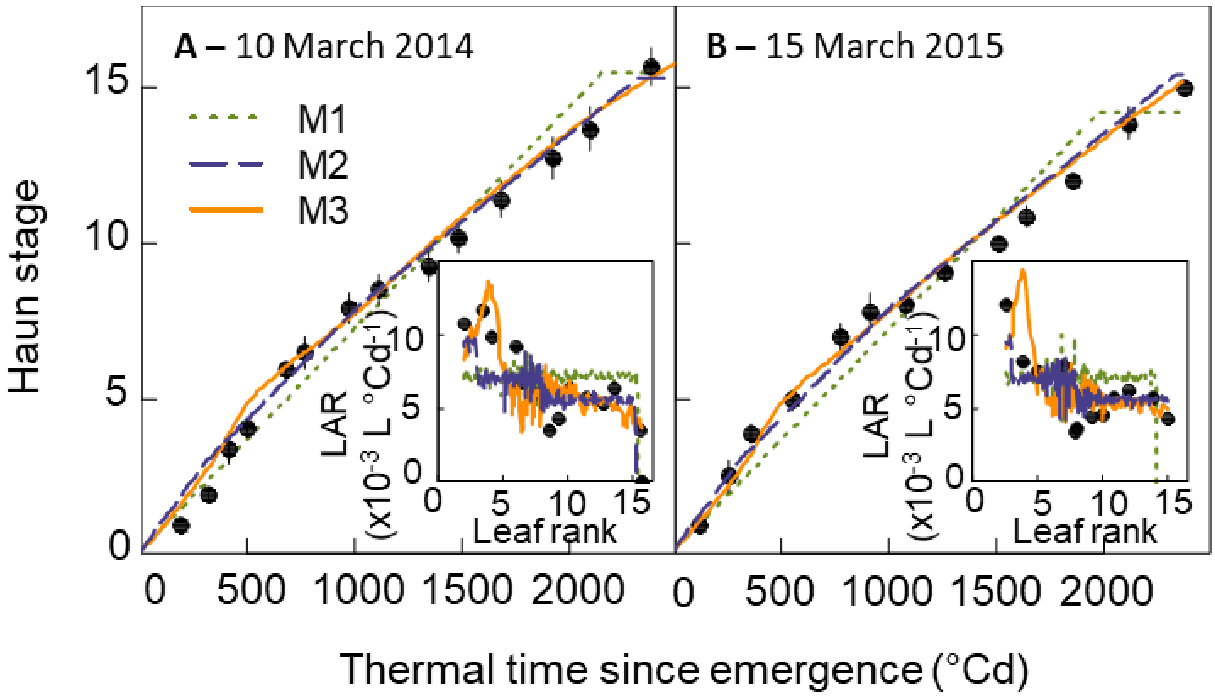

Figure S2. Relationship between observed (close circle) and simulated (lines) Haun stage and thermal time after emergence for the spring wheat cultivar Wakanui sown in the field at Leeston, New Zeland. Lines are simulations obtained with the wheat model *SiriusQuality* and using either a constant phyllochron (model M1, dotted green lines), a segmented linear model in thermal time corrected for the sowing date (model M2, dashed blue lines) or our new model (model M3, solid orange lines). Inset shows observed (closed circles) and simulated (lines) leaf appearance rate versus leaf rank. The thermal time was calculated using apex temperature and Eq. (1) for the observation and using simulated near-surface soil or canopy temperature and Eq. (1) for the simulated data (see Material and Methods). Data are mean ± 1 s.d. for n = 15 independent replicates.

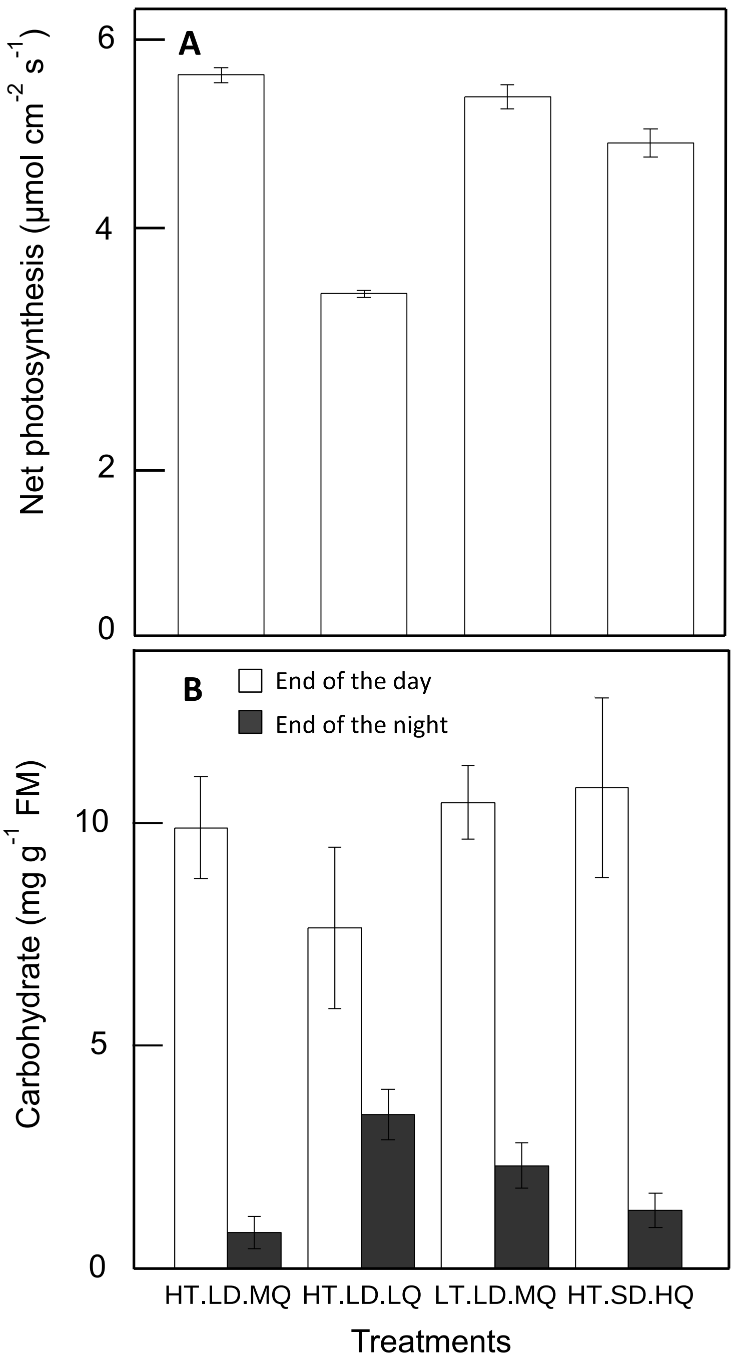

Figure S3. Net photosynthesis (A) and carbohydrate concentration (B) for the spring wheat cv. Paragon grown in controlled conditions with different combinations of temperature, irradiance, and photoperiod. Treatments are as in Figure 1. Data are mean ± 1 s.d. for *n* = 6 independent replicates.

References

Bos HJ, Neuteboom JH. 1998. Morphological analysis of leaf and tiller number dynamics of wheat (*Triticum aestivum* L.): Responses to temperature and light Intensity. *Annals of Botany* **81**, 131-139.

Cao W, Moss DN. 1989a. Daylength effect on leaf emergence and phyllochron in wheat and barley. *Crop Science* **29**, 1021-1025.

Cao W, Moss DN. 1989b. Temperature and daylength interaction on phyllochron in wheat and barley. *Crop Science* **29**, 1046-1048.

Cao W, Moss DN. 1989c. Temperature effect on leaf emergence and phyllochron in wheat and barley. *Crop Science* **29**, 1018-1021.

Gauch HG, Hwang JTG, Fick GW. 2003. Model evaluation by comparison of model-based predictions and measured values. *Agronomy Journal* **95**, 1442-1446.

Rickman RW, Klepper B, Peterson CM. 1985. Wheat seedling growth and developmental response to incident photosynthetically active radiation. *Agronomy Journal* **77**, 283-287.

Rousset M, Bonnin I, Remoué C, Falque M, Rhoné B, Veyrieras J-B, Madur D, Murigneux A, Balfourier F, Le Gouis J, Santoni S, Goldringer I. 2011. Deciphering the genetics of flowering time by an association study on candidate genes in bread wheat (*Triticum aestivum* L.). *Theoretical and Applied Genetics* **123**, 907-926.

Zheng B, Biddulph B, Li D, Kuchel H, Chapman S. 2013. Quantification of the effects of *VRN1* and *Ppd-D1* to predict spring wheat (*Triticum aestivum*) heading time across diverse environments. *Journal of Experimental Botany* **64**, 3747-3761.
